# Immediate intravenous epinephrine versus early intravenous epinephrine for in-hospital cardiopulmonary arrest

**DOI:** 10.1186/s12871-021-01346-1

**Published:** 2021-05-13

**Authors:** Abdullah Bakhsh, Maha Safhi, Ashwaq Alghamdi, Amjad Alharazi, Bedoor Alshabibi, Rajwa Alobaidi, Maryam Alnashri

**Affiliations:** 1 Department of Emergency Medicine, the King Abdulaziz University, Jeddah, 80215 Saudi Arabia; 2 Faculty of Medicine, the King Abdulaziz University, Jeddah, 80215 Saudi Arabia

**Keywords:** In-hospital cardiopulmonary arrest, Immediate epinephrine, ROSC, Survival to admission

## Abstract

**Background:**

Intravenous epinephrine has been a key treatment in cardiopulmonary arrest since the early 1960s. The ideal timing for the first dose of epinephrinee is uncertain. We aimed to investigate the association of immediate epinephrine administration (within 1-min of recognition of cardiac arrest) with return of spontaneous circulation (ROSC) up to 24-h.

**Methods:**

This was a multicenter retrospective analysis of patients who underwent cardiopulmonary resuscitation. We included the following patients: 1) ≥18 years-old, 2) non-shockable rhythms, 3) received intravenous epinephrine during cardiopulmonary resuscitation, 4) witnessed in-hospital arrest and 5) first resuscitation attempt (for patients requiring more than one resuscitation attempt). We excluded patients who suffered from traumatic arrest, were pregnant, had shockable rhythms, arrested in the operating room, with Do-Not-Resuscitate (DNR) order, and patient aged 17 years-old or less.

**Results:**

A total of 360 patients were included in the analysis. Median age was 62 years old and median epinephrine administration time was two minutes. We found that immediate epinephrine administration (within 1-min) is associated with higher rates of ROSC up to 24-h (OR = 1.25, 95% CI; [1.01–1.56]), compared with early epinephrine (≥2-min) administration. After adjusting for confounding covariates, earlier administration of epinephrine predicted higher rates of ROSC sustained for up to 24-h (OR 1.33 95%CI [1.13–1.55]).

**Conclusions:**

Immediate administration of epinephrine in conjunction with high-quality CPR is associated with higher rates of ROSC.

## Introduction

Epinephrine has been a key treatment in advanced cardiac life support (ACLS) since cardiopulmonary resuscitation (CPR) guidelines were first published in early 1960s [1]. The alpha-agonist effect of epinephrine causes increase in aortic diastolic pressure, augmenting the coronary and cerebral blood flow [1]. Various studies have shown that the use of epinephrine is associated with increase in return of spontaneous circulation (ROSC) rates because of its alpha-agonistic effects [2, 3]. However, there is uncertainty about its effect on survival to hospital discharge and neurologic recovery. [4–6] Epinephrine may produce a mismatch between oxygen demand and delivery which could result in lactic acidosis. Moreover, the vasoconstrictor effects may prolong ischemia in some tissues. This has been seen particularly in Swine brain [7, 8]. In fact, direct visualization of brain capillaries reveals constricted microvessels, with little or no perfusion to brain tissue. This effect is due to the alpha-1 agonist effects of epinephrine [1, 7, 8].

Goto et al investigated the pre-hospital use of intravenous epinephrine and its effect on ROSC and neurological outcomes in large Japanese database. When given within 9-min of cardiac arrest, epinephrine is associated with higher rates of ROSC compared to patients who did not receive epinephrine. However, neurologic outcomes were poorer in patients receiving epinephrine at any given time during cardiac arrest [5].

The American Heart Association (AHA) recommends giving epinephrine as early as possible then every 3–5 min thereafter [9]. Various trials suggest a time-dependent effect of epinephrine on outcomes of CPR; earlier administration of intravenous epinephrine may improve outcomes [10, 11]. However, previous studies have shown that delays in the administration of epinephrine are common in clinical practice. Thus, this is -found to be associated with worse outcomes in both adults and children [12, 13].

A study in 2014 utilized the AHA’s Get With The Guidelines-Resuscitation (GWTG-R) database which included in-hospital arrest across 570 American hospitals. This study showed that earlier administration of epinephrine in patients with non-shockable cardiac arrest rhythms was associated with increased ROSC and survival. Moreover, a stepwise decrease in survival with every increase in interval of time to epinephrine [12]. Another study in 2016 also including in-hospital cardiac arrest from the GWTG-R database in US hospitals found improvements in ROSC and survival with functional recovery with timely administration of epinephrine [14]. A recent study in 2019 which examined the GWTG-R database in US hospitals revealed that delays in intravenous epinephrine administration was associated with lower survival [15].

The lack of rigorous experimental studies on the clinical outcomes associated with epinephrine has led the resuscitation community to continue recommending epinephrine in cardiac arrest. However, the PARAMEDIC-2 trial might change the way clinicians think about epinephrine. The study conducted by Perkins et al. in the United Kingdom, included 8014 patients who underwent out-of-hospital cardiac arrest. Patients were randomized to receive either epinephrine (*n* = 4015) or placebo (*n* = 3999). Primary outcome was 30-day survival, and secondary outcomes were survival to hospital discharge and neurologically intact status. The authors found that the administration of epinephrine increased 30-day survival rates (3.2% in the epinephrine group, compared to 2.4% in the placebo group). However, a larger proportion of patients in the epinephrine group were neurologically devastated, with modified Rankin scores of 4–5 (31% in the epinephrine group, compared to 17.8% in the placebo group). This result demonstrated a lack of overall improvement neurologically in the epinephrine group, despite the higher rate of overall survival [16, 17]. The authors postulate that despite the improvement in the macrovascular cerebral perfusion pressures, epinephrine may cause microvascular ischemia in the brain, thereby worsening anoxic brain injury. A key finding of the PARAMEDIC-2 trial is that the mean time to epinephrine administration was 21.5 min. The significant difference in time frame precludes the generalizability to the in-hospital setting.

Although epinephrine can increase the likelihood of achieving ROSC, the optimal time of epinephrine is still uncertain [17]. It seems intuitive that immediate administration of epinephrine with cardiopulmonary resuscitation will maintain perfusion and therefore, reduce bad outcomes. Our primary objective is to compare the association of immediate administration of epinephrine (within 1-min) with early administration of epinephrine (≥2-min) in sustained ROSC (≥20-min – 24-h) in non-shockable in-hospital cardiopulmonary arrest.

## Materials and methods

### Study design

This was a multicenter retrospective study conducted in Jeddah, Saudi Arabia. This study was approved by the department of research and studies at the Ministry of Health in Saudi Arabia (registration no. H-02-J-002, approval no. A00440). Informed consent was waived due to the retrospective nature of the study. Researchers retrospectively collected data from cardiac arrest flowsheets from each site between January 2016 and January 2017. We included patients 1) ≥18 years-old, 2) non-shockable rhythms, 2) received intravenous Epinephrine during cardiopulmonary resuscitation, 3) witnessed in-hospital arrest, 4) only the first resuscitation attempt (for patients requiring more than one attempt) and 5) chest compression started within 1-min of recognition. We excluded patients who suffered traumatic arrest, were pregnant, had shockable rhythms, in the operating room, had a Do-Not-Resuscitate (DNR) order, and parents aged 17 years-old or less.

### Study setting

It is standard procedure in the Kingdom of Saudi Arabia to activate code blue in a hospital to mobilize the resuscitation team. The definition of cardiopulmonary arrest at each site is the cessation of cardiac function manifesting as a non-palpable carotid pulse. Upon recognition, personnel are required to call for assistance and immediately start chest compressions. Each site requires all medical staff to be certified in basic life support at the minimum. Code blue team is typically composed of a critical care physician, an anesthesiologist, an intensive care unit nurse, in addition to the primary team and staff nurse from the location of arrest. Team members responding to code blue activations are certified in advanced cardiac life support to ensure standardized treatment. The composition of the resuscitation team may differ from one institution to another based on staff expertise and patient needs. Epinephrine is always administered by the code blue team nurse at the discretion of the code blue team leader. Cardiopulmonary arrest flow sheets may differ in format between institutions; however, all contain the key information according to the Utstein guideline [18] for documentation. Data included on the flow sheet include: patient information and demographics, time and date of arrest, location of arrest, available team members and time of response, initial and subsequent rhythms, medications and doses administered, type of airway device placed, presence or absence of return of spontaneous circulation, and post-arrest vital signs. The data is entered in real time during the resuscitation by a nurse designated and trained in documentation.

### Definitions

Time to epinephrine was defined as the interval in minutes from recognition of loss of pulse to the first bolus of 1 mg intravenous epinephrine. A registered nurse is delegated the task of documenting time intervals during resuscitation in all resuscitations. Each center provides special training for documenting events during resuscitation to ensure standardization. We defined sustained ROSC as sustained return of pulse for at least 24-h regardless of subsequent events during the 24-h period.

### Statistical analysis

Descriptive statistics were used to characterize the study population. The Pearson’s Chi Square test or independent T-test was used to compare variables between the immediate (within 1-min) epinephrine group with the early (> 2-min) epinephrine group. Furthermore, we constructed a logistic regression model analyzing the primary outcome of ROSC as an independent categorical variable according to the time of epinephrine administration to determine potential associations between confounding variables including age, gender, race, initial rhythm (PEA/asystole), CPR duration, endotracheal intubation during CPR (yes/no), institution (hospital A, B, C), level of care (emergency department, intensive care unit, floor), and diagnosis category (cardiac, respiratory, central nervous system, gastrointestinal, metabolic). Selection of these variables were planned a priori and based on previous studies. A *p*-value of less than 0.05 was considered significant. We reported the unadjusted and adjusted odds ratio with 95% confidence interval for statistical testing. Analyses were performed using the IBM SPSS statistical package for Windows, version 25.0, SPSS, Inc., Chicago, IL, USA.

## Results

**Fig. 1:**
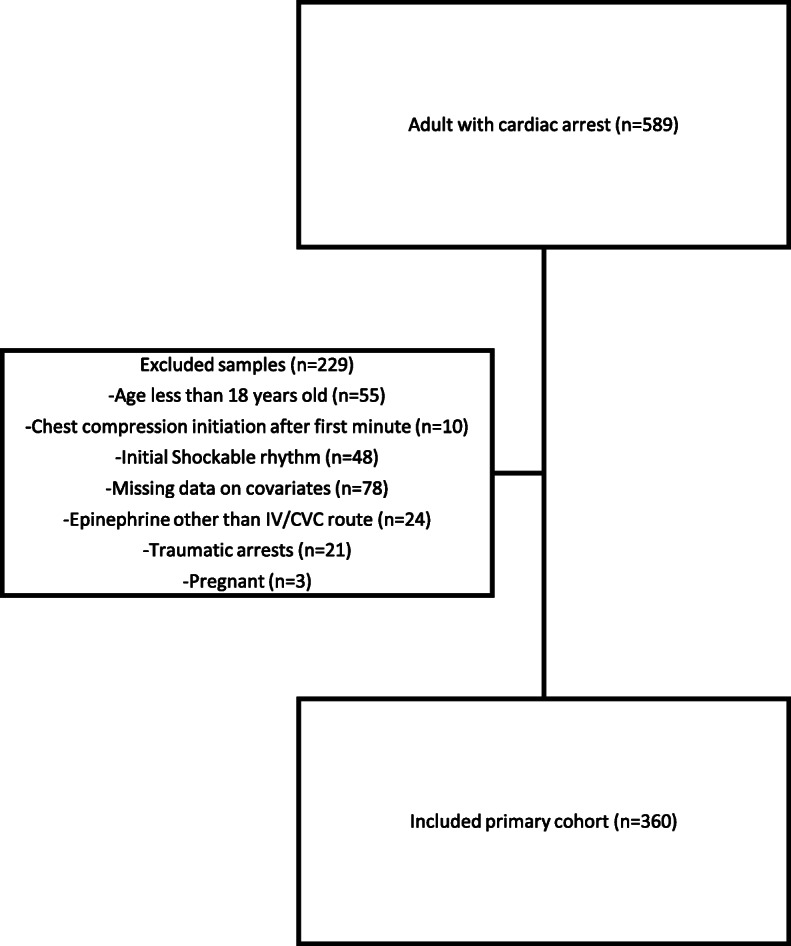
Inclusion and exclusion of patients’ flowchart

A total of 589 charts from three different sites were screened (Fig. 1). After-applying the exclusion criteria, only 360 patients were included for analysis. Table 1 shows the baseline characteristics of the patients. The median time to epinephrine administration was 2.00 min (IQR 3-min) and median CPR duration was 20:00 min (IQR 16:45 min). Sustained ROSC (20-min – 24-h) was achieved in 95 patients (26.40%).

**Table 1:** Baseline characteristics of patients

	All patients (***n*** = 360)	Epinephrine 0–1 min (***n*** = 177)	Epinephrine ≥ 2 min (***n*** = 183)	***P***-value
**Median age in years (IQR)**	62 (26)	62 (25.50)	62 (25)	0.90
**Male n(%)**	220 (61.1%)	113 (63.8%)	107 (58.5%)	0.29
**Female n(%)**	140 (38.9%)	64 (36.2%)	76 (41.5%)	
**Saudi Arabian**	194 (53.9%)	94 (53.1%)	100 (54.6%)	0.77
**Non-Saudi**	166 (46.1%)	83 (46.9%)	83 (45.4%)	
**Median CPR duration in minutes (IQR)**	20:00 (16:45)	16:00 (15:00)	20:00 (15:00)	0.70
**Median epinephrine time in minutes (IQR)**	2:00 (3)	0:00 (0)	3:00 (3)	< 0.01
**PEA n(%)**	132 (36.7%)	95 (53.7%)	37 (20.2%)	< 0.01
**Asystole n(%)**	228 (63.3%)	82 (46.3%)	146 (79.8%)	
**Intubation during CPR n(%)**	199 (55.3%)	94 (53.1%)	105 (57.4%)	0.41
**No intubation during CPR n(%)**	161 (44.7%)	83 (46.9%)	78 (42.6%)	
**ROSC n(%)**	95 (26.4%)	55 (31.1%)	40 (21.9%)	0.04
**Respiratory n(%)**	132 (36.7%)	53 (29.9%)	79 (43.2%)	< 0.01
**Cardiac n(%)**	95 (26.4%)	60 (33.9%)	35 (19.1%)	
**CNS n(%)**	48 (13.3%)	22 (29.9%)	26 (14.2%)	
**Metabolic n(%)**	43 (11.9%)	26 (14.7%)	17 (9.3%)	
**GI n(%)**	43 (11.7%)	16 (9.0%)	26 (14.2%)	
**ICU n(%)**	172 (47.8%)	83 (46.9%)	89 (48.6%)	0.01
**ED n(%)**	117 (32.5%)	68 (38.4%)	49 (26.8%)	
**Floor n(%)**	71 (19.7%)	26 (14.7%)	45 (24.6%)	
**Hospital A n(%)**	168 (46.7%)	76 (42.9%)	92 (50.3%)	0.10
**Hospital B n(%)**	98 (27.2%)	46 (26.0%)	52 (28.4%)	
**Hospital C n(%)**	94 (26.1%)	55 (31.1%)	39 (21.3%)	

Immediate (within 1-min) epinephrine administration was observed in 166 patients (46.10%), whereas early (≥2-min) epinephrine administration was seen in 144 patients (53.90%).

Our results reveal that the immediate administration of intravenous epinephrine is associated with statistically higher rates of ROSC (20-min – 24-h); 15.3% vs. 11.1% (*p* = 0.04).

**Fig. 2:**
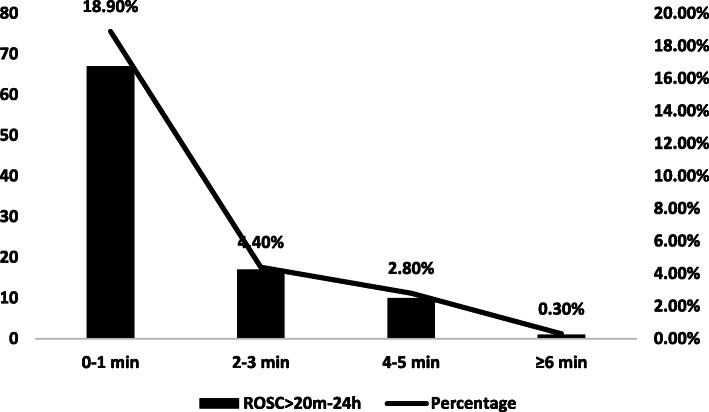
Association Between Timing of First Dose of Epinephrine With Sustained Return of Spontaneous Circulation (≥20-min but < 24-h)

A graphical illustration (Fig. 2) shows a stepwise decrease in sustained ROSC (20-min – 24-h) with every 1-min delay in epinephrine administration: 18.90% showed sustained ROSC when receiving their first dose of epinephrine between 0 and 1 min. This was decreased to 4.40% when the first dose of epinephrine was received between 2 and 3 min, to 2.80% when epinephrine received between 4 and 5 min and down to 0.30% when the first dose was administered at 6-min or later (*P* < 0.01).

After adjusting for potential covariates, each minute delay in the administration of epinephrine was associated with 33% decrease in the odds of sustained ROSC (20-min – 24-h) (OR 1.33 95%CI [1.13–1.55]) (Table 2). Moreover, the same regression model showed that initial rhythm and airway placement had an independent effect on ROSC. Asystole was associated with lower odds of sustained ROSC (OR 1.96 95%CI [1.14–3.39]). Patients who did not get intubated during CPR were associated with higher odds of sustained ROSC (OR 0.50 95%CI [0.30–0.83]).

**Table 2:** Adjusted Odds Ratios for Potential Confounding Factors

	P-value	aOR [95% CI]
**First dose epinephrine**	< 0.01	1.33 [1.38–1.55]
**Rhythm**	0.01	1.96 [1.14–3.39]
**Airway**	< 0.01	0.50 [0.30–0.83]

## Discussion

Our findings reveal that immediate administration of intravenous epinephrine is associated with increased sustained return of spontaneous circulation (ROSC) (20-min – 24-h). There had been some controversy on the ability of epinephrine to increase ROSC rates. However, there is a strong evidence from large number of clinical studies that epinephrine use improves the chances of ROSC, but does not benefit survival [19–21]. Notably, some studies suggest that epinephrine might actually worsen the neurologic outcome with increasing cumulative dose of epinephrine [22]. Early epinephrine administration is practically achievable for in-hospital cardiac arrest as opposed to out-of-hospital settings. Our study reveals that median epinephrine administration time is 2.00 min. A study by Hansen at al [23]. conducted a secondary analysis on 26,755 patients in the out-of-hospital setting. A 10-min cut off time from emergency medical services (EMS) arrival to epinephrine administration was applied. The majority received epinephrine > 10 min from EMS arrival (54.2%). The highest survival to discharge was noted when epinephrine was given before 4 min, which occurred in only 7% of patients. Moreover, each additional minute of time from EMS arrival to epinephrine was associated with 4% decrease in odds of survival to hospital discharge (OR 0.96; 95%CI 0.95–0.98). However, there are profound differences in patients’ characteristics, underlying etiology, treatment and timing and outcomes between patients in and out of hospital.

Donnino et al. [12] conducted a post hoc analysis of prospectively collected data in a large multicenter registry of in-hospital cardiac arrests (Get With The Guidelines-Resuscitation). They included 25,095 patients from 570 hospitals with asystole (55%) or pulseless electrical activity (45%). The median time to epinephrine administration was three minutes (interquartile range 2–4). Survival to 24-h occurred in 6280 (27%) patients, but only 2603 (10%) survived to discharge. A stepwise decrease in survival to discharge with additional minute of first administration of epinephrine was also observed: 929 (12%) survived when epinephrine was given in the first minute, 392 (12%) in the second minute, 305 (11%) in the third minute, 208 (9%) in the fourth minute, 335 (10%) in the fifth minute, 124 (10%) in the sixth minute, and 310 (7%) in the seventh minute or later (*P* < 0.001). The results of our study were consistent with the stepwise decrease in ROSC with every minute delay in epinephrine administration however, our primary outcome with sustained ROSC for up to 24-h. We used a cutoff of 1-min for first epinephrine administration. This was used due to the time-sensitive interventions required during the low-flow state to maintain coronary and cerebral perfusion without interruption. Results of this study show that immediate epinephrine administration is associated with higher rates of ROSC (20-min – 24-h) (OR 1.93; 95%CI 1.58–2.36) when compared with early epinephrine (≥2-min). Additionally, Fig. 2 demonstrates a sharp decrease in ROSC from 18.90% when epinephrine was administered between 0 and 1 min to 4.40% when epinephrine was administered between 2 and 3 min. Therefore, the number of patients who need to be treated with epinephrine within 1-min to achieve one patient with ROSC (20 min-24 h) is 7.

Interestingly, an initial rhythm of PEA and avoiding intubation during CPR independently carry a higher likelihood of sustained ROSC when compared with asystole and intubation during CPR. These findings are consistent with previous studies [24–26]. PEA generally carries better prognosis than asystole [24, 25]. However, the insertion of an endotracheal tube during CPR may hinder from more critical actions such as chest compressions or the early administration of epinephrine. A retrospective study by Anderson et al. examined the GWTG-R registry and found that patients intubated in the first 15-min of cardiac arrest had lower survival compared to those intubated after the first 15-min (RR 0.75 95%CI [0.73–0.76]) [26].

While the American Heart Association (AHA) recommend immediate and uninterrupted chest compressions to maintain coronary and cerebral perfusion, there is no current strong recommendations on the timing of first epinephrine administration nor any recommendation on a maximum dose [9]. In fact, the latest AHA guidelines recommend that epinephrine should be administered as early as possible then every 3–5-min thereafter [9]. The physiologic rationale for early epinephrine administration is strong [1–3]. The combination of immediate high-quality chest compression and immediate epinephrine administration could potentially result in better outcomes. Although, the results of our study encourage immediate epinephrine administration, they question the benefit of epinephrine after a certain amount of time.

### Limitations

This was a retrospective analysis. This limitation may have been addressed by applying regression models. However, it is possible that unmeasured confounding factors still exist. Data represents experiences from three different sites which may have also affected the results. Moreover, we were unable to assess the quality of cardiopulmonary resuscitation in each case and investigate how it affected the results of our study. This may limit the generalizability of our results.

## Conclusion

Immediate epinephrine administration is associated with better rates of ROSC for up to 24-h for in-hospital cardiopulmonary arrests with non-shockable rhythms. This is achievable in the in-hospital setting. Therefore, we encourage initiating immediate CPR in conjunction with immediate epinephrine administration. Larger studies are required to investigate on the benefits of immediate epinephrine administration.

## Data Availability

Data are available upon request from the corresponding author.
